# Pathogenic Yeasts Deploy Cell Surface Receptors to Acquire Iron in Vertebrate Hosts

**DOI:** 10.1371/journal.ppat.1003498

**Published:** 2013-08-29

**Authors:** James W. Kronstad, Brigitte Cadieux, Won Hee Jung

**Affiliations:** 1 The Michael Smith Laboratories, Department of Microbiology and Immunology, University of British Columbia, Vancouver, British Columbia, Canada; 2 Department of Systems Biotechnology, Chung-Ang University, Anseong, Republic of Korea; Duke University Medical Center, United States of America

## Introduction

Brown et al. (2012) [1] recently highlighted the growing threat that fungal pathogens pose for humans, as well as the pressing need for additional antifungal drugs and efficacious vaccines. In this context, the process of iron acquisition presents compelling opportunities to prevent or treat fungal diseases because iron is an essential nutrient for pathogen proliferation in vertebrate hosts. Fungi and other pathogens must deploy competitive uptake mechanisms to steal iron from host sources and overcome the iron sequestration associated with nutritional immunity [2]. These extracellular and surface uptake functions may provide readily accessible targets for drugs to block iron uptake, and iron transporters might also be exploited to introduce antifungal agents into fungal cells [3], [4]. Additionally, extracellular or exposed transporters may be useful vaccine targets to block iron uptake and pathogen proliferation.

**Figure 1:**
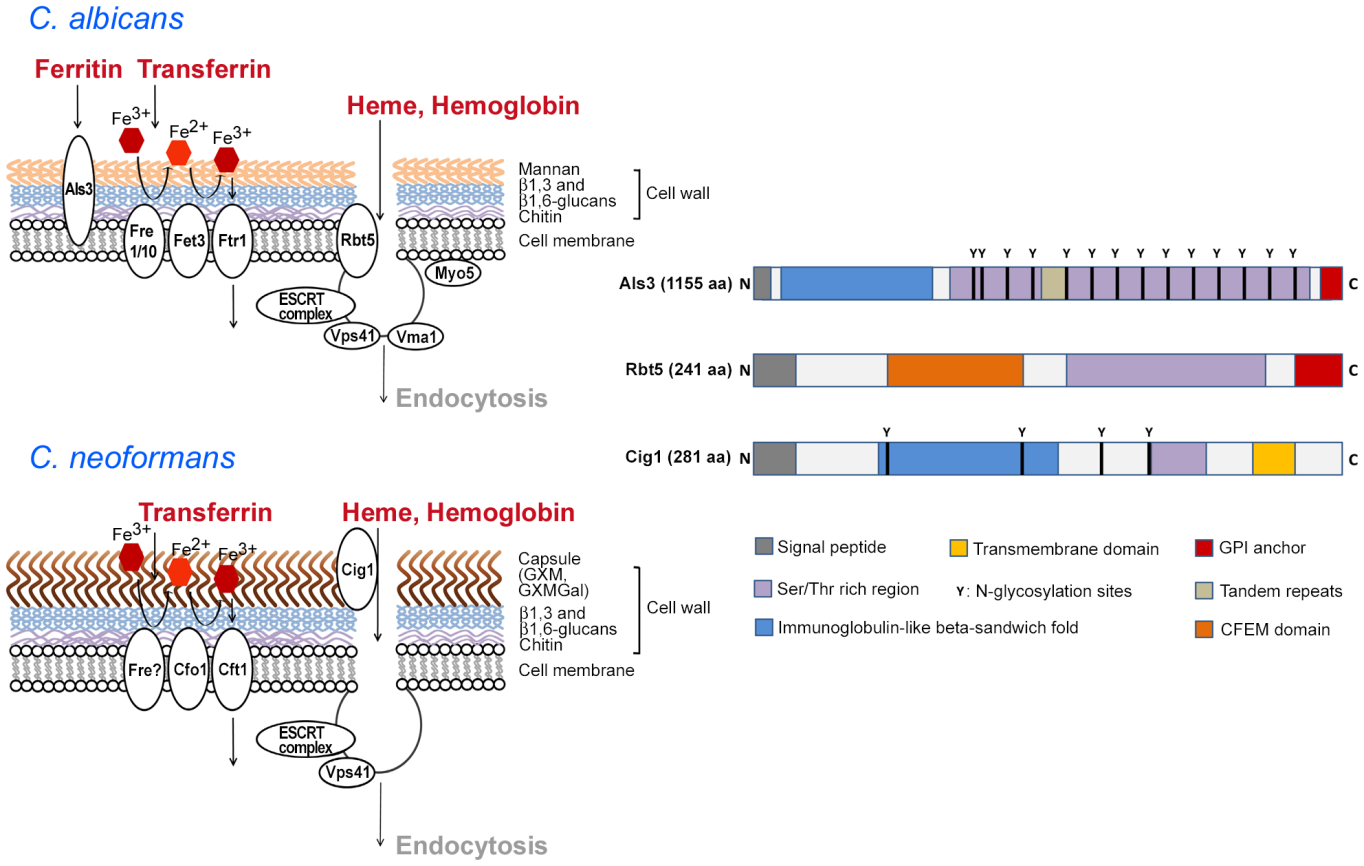
Cell surface functions for iron acquisition in *C.* *albicans* and *C. neoformans*. The diagrams on the left show the polysaccharides and iron-related uptake functions at the fungal cell surface. For *C. albicans*, the iron-uptake proteins include the ferritin-binding protein Als3, ferric reductases (Fre1/10), a multicopper oxidase (Fet3), and an iron permease (Ftr1) [8]–[10]. The components of a heme uptake pathway include the receptor Rbt5 and some of the proteins involved in internalization [18], [19]. These proteins are depicted schematically as contributing to endocytosis of heme and/or hemoglobin. For *C. neoformans*, surface polysaccharides are shown for the capsule (GXM, GXMGal) and the cell wall, and iron-uptake functions include putative ferric reductase activity (Fre), a multicopper oxidase (Cfo1), and an iron permease (Cft1) [7], [37]. The role of Cig1 and known or hypothesized functions for endocytosis are also shown in a schematic depiction [27], [30]. The diagrams on the right present the structures of Als3 [10], Rbt5 [17]–[19], and Cig1 ([27]; B. Cadieux, unpublished) to depict shared and distinct domains. Note that the polypeptides are not draw to scale and the actual amino acid length of each is indicated. Molecular modeling suggests that the immunoglobulin-like fold of Als3 may interact with cadherins [10]. However, the structural features that contribute to iron acquisition have not been defined for any of the proteins.

Mechanisms of iron acquisition have been well characterized in many microbial pathogens, and information is rapidly accumulating for fungal pathogens [5]–[9]. Fungi generally acquire iron by several mechanisms including: 1) the production and uptake of siderophores; 2) the use of a ferroxidase-iron permease complex for high-affinity uptake; 3) the transport of ferrous iron; and 4) the acquisition of iron from heme and hemoglobin [7]–[9]. Reductases in the plasma membrane and secreted reductants facilitate the reduction of ferric iron to ferrous iron for high- or low-affinity uptake [7]–[9]. The exploration of these mechanisms in fungal pathogens has revealed intriguing connections between cell surface molecules (cell wall and secreted proteins, capsular polysaccharide, and biofilms) and functions for iron acquisition from vertebrate sources (Figure 1A, B). Here we highlight these connections for the pathogenic yeasts *Candida albicans* and *Cryptococcus neoformans*. We also discuss the extent to which the newly identified functions add depth to our understanding of iron acquisition by fungi and illustrate the complex integration of iron sensing and virulence.

## Cell Surface Proteins Link the Use of Ferritin, Heme, and Hemoglobin with Biofilm Formation in *C. albicans*

The adhesin Als3 and the CFEM domain protein Rbt5 are two notable examples of proteins that link cell surface activities related to morphogenesis and/or biofilm formation with iron acquisition in *C. albicans* (Figure 1C). Als3 is a hypha-specific surface protein that functions as an adhesin for epithelial and endothelial cells, and that also mediates adherence to extracellular matrix proteins (reviewed in [10]). The ability of *C. albicans* to switch its morphology between yeast and hyphal forms is important for virulence, as is the ability of the fungus to form biofilms on implanted medical devices. Als3 contributes to virulence in mouse models of candidiasis, although the impact of the protein depends on the method of inoculation and the immune status of the host [10]. Expression of *ALS3* is transcriptionally controlled in a complex manner by regulators of the yeast-hyphal transition, by the major regulator of biofilm formation Bcr1, and by the alkaline response transcription factor Rim101 [10]–[14].

Als3 is an interesting multifunctional protein because, in addition to binding to and provoking endocytosis of the fungus by host cells, it also plays a role in biofilm formation and it binds the host iron-storage protein ferritin. Specifically, Almeida et al. (2008) [15] found that *C. albicans* can use ferritin as an iron source at physiological pH, and that Als3 is required for this process. Ferritin is bound by hyphal cells that express Als3 (but not by yeast cells), and deletion of the *ALS3* alleles eliminates both binding and growth on ferritin. The ferritin binding by hyphae is also observed when these cells invade epithelial cells *in vitro*, and Als3 is required for *C. albicans* to damage these cells [15]. Overall, Als3 functionally links biofilm formation, the yeast-hyphal transition, and the use of a specific host iron source.

The second example of the connection between biofilm formation, morphology, and *C. albicans* iron acquisition involves Rbt5, an O-mannosylated, glycosylphosphatidylinositol (GPI)-anchored protein (Figure 1C) [16]–[18]. Rbt5 is located in the plasma membrane and the cell wall, the protein binds heme and hemoglobin, and deletion of *RBT5* reduces the use of these host iron sources by *C. albicans* [16]–[18]. *RBT5* expression is induced by iron limitation and negatively regulated by the Tup1 regulator of morphology [17], [19]. Related genes encoding CFEM domain proteins are present in *C. albicans*, and these include *RBT51/PGA10*, *CSA1*, *CSA2*, and *PGA7*. Rbt51 also participates in hemoglobin binding and, in fact, the protein was originally identified in a screen for *C. albicans* genes that allowed *S. cerevisiae* to use iron from hemoglobin [17]. Weissman et al. (2008) [18] exploited this property of Rbt51 to identify mutations in *S. cerevisiae* that blocked hemoglobin use. The identified functions included subunits of the vacuolar ATPase, components of the ESCRT system, HOPS complex proteins and t-SNAREs, and several other functions. Notably, the ESCRT and HOPS complexes, and the t-SNAREs, contribute to endocytosis. An examination of *C. albicans* mutants with defects in the corresponding genes confirmed that many of these functions played roles in iron use from hemoglobin (e.g., Vma11, Vps41, ESCRT complex components, Myo5). Overall, these studies revealed an endocytic pathway for heme/hemoglobin internalization and trafficking to the vacuole for processing.

Similar to Als3, mutants lacking Rbt5, Rbt51, or the related protein Csa1 form thinner and more fragile biofilms with less extracellular matrix compared with the wild-type strain [20], [21]. These mutants also displayed changes in their cell surface as examined microscopically and by measurements of cell surface hydrophobicity. Interestingly, conserved transcriptional regulation by the biofilm regulator Bcr1 is observed for three CFEM genes in *C. albicans* (*RBT5*, *PGA7*, and *CSA1*) and for three genes (*CFEM2*, *CFEM3*, and *CFEM6*) in the related pathogen *Candida parapsilosis* [22]. However, these proteins have divergent roles in that the three CFEM proteins are not involved in biofilm formation in *C. parapsilosis* [22].

## Links between the Cell Surface and Heme Uptake in *C. neoformans*

The role of extracellular proteins in iron acquisition and connections with the cell surface extends to *C. neoformans*. In this fungus, the *CIG1* gene was identified by transcriptional profiling as the most abundant transcript in iron-starved cells, and the protein had previously been identified as an extracellular cytokine-inducing glycoprotein (Figure 1C) [23], [24]. Glycoproteins (mannoproteins) associated with the polysaccharide capsule have been proposed as vaccine candidates for *C. neoformans* [24], [25]. The polysaccharide capsule is a major virulence factor for *C. neoformans*, and it has long been known that the fungus produces a large capsule upon iron deprivation and a small capsule in iron-replete conditions [26]. Interestingly, disruption of the *CIG1* gene in a strain with the D capsular serotype resulted in altered capsule regulation by iron [23]. More recently, it was found that deletion of *CIG1* in both serotype A or D strains resulted in delayed growth on heme as the sole iron source ([27]; B. Cadieux, unpublished). This growth delay was observed at physiological pH but not at acidic pH, and this result was consistent with the observation of O'Meara et al. (2010) [28] that the pH-responsive transcription factor Rim101 activates expression of *CIG1*. Furthermore, a *rim101* mutant also had delayed growth on heme [27].

Cig1 may participate in an uptake mechanism that involves heme accumulation at the cell surface because recombinant Cig1 binds heme, suggesting that it may be a hemophore [27]. A hemophore would potentially allow access to heme, which contains the majority of the iron present in vertebrate hosts. A role in heme uptake is supported by the finding that a *cig1* mutant displays reduced susceptibility to non-iron metalloporphyrins such as gallium protoporphyrin IX (Ga-PPIX). In bacterial pathogens, these compounds rely on heme uptake to cause toxicity, and they serve as reagents to distinguish heme uptake from processing at the cell surface [29]. Functions downstream of Cig1 that appear to participate in heme uptake may include components of the ESCRT pathway because the ESCRT-I protein Vps23 is required for heme uptake, susceptibility to Ga-PPIX, capsule attachment, and virulence [30]. In this case, ESCRT complexes may contribute to endocytosis and intracellular trafficking of heme in *C. neoformans*, as was found in *C. albicans*.

A mutant lacking Cig1 was equivalent in virulence to the wild-type parental strain in a mouse inhalation model of cryptococcosis, and a contribution to virulence was only revealed when the *cig1* mutation was combined with a mutation in the *CFO1* gene encoding a ferroxidase for high-affinity uptake. This latter finding reinforces a key conclusion about iron acquisition and virulence: pathogens appear to have multiple redundant mechanisms to acquire iron because of the critical requirement for the metal and the need to overcome host defense by sequestration. However, it is curious that Cig1 makes a positive contribution to virulence while loss of its regulator, Rim101, results in hypervirulence. O'Meara et al. (2013) [31] recently investigated the virulence contribution of Rim101 in more detail and found that the protein regulates the composition of the cell wall, leading to increased stimulation of the host immune response. Part of the surface change in a *rim101* mutant includes release of approximately four times more capsule polysaccharide into the culture medium than the wild-type strain, and this may result from changes in the cell wall that reduce attachment. The regulation of cell wall functions by Rim101 has been observed previously in other fungi [14].

## Perspective

The integration of iron sensing, virulence factor expression, and biofilm formation has been previously documented in bacterial pathogens such as *Pseudomonas aeruginosa* [32]. The studies outlined above reveal that the pathogenic yeasts *C. albicans* and *C. neoformans* also have intriguing connections between mechanisms to sense and regulate the response to host conditions, acquire iron from host sources, and remodel cell surface features. In particular, a growing list of proteins have been found to contribute to iron acquisition and to also influence non-protein surface components such as cell wall glucans, capsular polysaccharides, and perhaps the extracellular matrix in biofilms. These contributions may simply indicate that the proteins have more than one function or there may be underlying functional implications. In the latter case, it is interesting to speculate that one role of the capsule polysaccharide in *C. neoformans* and the biofilm-associated extracellular matrix in *C. albicans* may be to sequester iron and other nutrients close to the cell surface to facilitate uptake. This idea is supported by a growing body of evidence that extracellular polysaccharides bind small molecules. For example, β-1,3 glucans in *C. albicans* biofilms bind antifungal drugs, and glucuronoxylomannan in the *C. neoformans* capsule binds divalent cations [33]–[35]. Of course, it is also possible that pathogens must coincidently express iron-uptake functions to counteract possible interference of polysaccharides with iron uptake. The challenge now is to find approaches to test the positive and (potentially) negative contributions of the extracellular matrix in *C. albicans* and the polysaccharide capsule in *C. neoformans* for acquisition of iron and other nutrients. The ongoing identification of mutants with defects in polysaccharide synthesis, modification, and delivery will provide opportunities to meet this challenge [36], [37]. Furthermore, more detailed studies are needed to test the relationship between iron and biofilm formation by *C. albicans*. Hameed et al. (2008) [38] suggested that iron deprivation does not influence biofilm formation because the wild-type strain and an *ftr1* (iron permease) mutant were still able to form biofilms in the presence of an iron chelator. However, more work is needed because the mutant may not have been sufficiently starved for iron given that it still grew under the conditions tested.

Finally, we note that the iron-acquisition proteins are particularly good candidate vaccine targets because of their contributions to more than one virulence-related function. In this context, the CFEM proteins may be useful vaccine targets for *C. albicans* because Mochon et al. (2010) [39] found Rbt5 and Csa1 (and a ferric reductase) among 33 antigens recognized by sera from convalescent candidemia patients. Als3 is also a promising vaccine candidate for *C. albicans* because recombinant Als3 protects immunocompetent mice from candidiasis [10], [16].
